# Cold shock protein RBM3 is upregulated in the autophagy-deficient brain

**DOI:** 10.17912/micropub.biology.000695

**Published:** 2022-12-08

**Authors:** Junnosuke Nakamura, Takuma Aihara, Tomoki Chiba, Fuminori Tsuruta

**Affiliations:** 1 Graduate School of Life and Environmental Sciences, University of Tsukuba, 1-1-1 Tennodai, Tsukuba, Ibaraki 305-8577, Japan; 2 Master's and Doctoral Program in Biology, Faculty of Life and Environmental Sciences, University of Tsukuba, 1-1-1 Tennodai, Tsukuba, Ibaraki 305-8577, Japan; 3 Ph.D. Program in Human Biology, School of Integrative and Global Majors, University of Tsukuba, 1-1-1 Tennodai, Tsukuba, Ibaraki 305-8577, Japan; 4 Ph.D. Program in Humanics, School of Integrative and Global Majors, University of Tsukuba, 1-1-1 Tennodai, Tsukuba, Ibaraki 305-8577, Japan; 5 Master's and Doctoral Program in Neuroscience, Graduate School of Comprehensive Human Sciences, University of Tsukuba, 1-1-1 Tennodai, Tsukuba, Ibaraki 305-8577, Japan

## Abstract

Neural autophagy plays an important role in regulating protein quality control, brain homeostasis, and body temperature. However, the mechanism that links a defect in autophagy to body temperature has not been elucidated. Here, we report that RNA binding motif protein 3 (RBM3) is a potential candidate that regulates body temperature. We found that the body temperatures of *Nestin-Cre* ; *Atg7 ^f/f^* conditional KO (cKO) mice were lower than that of wild-type (WT) mice. Moreover, RBM3 was upregulated in the *Nestin-Cre* ; *Atg7 ^f/f^* brain. These data suggest that RBM3 is an implicit target that maintains body temperature influenced by neural autophagy.

**Figure 1.**
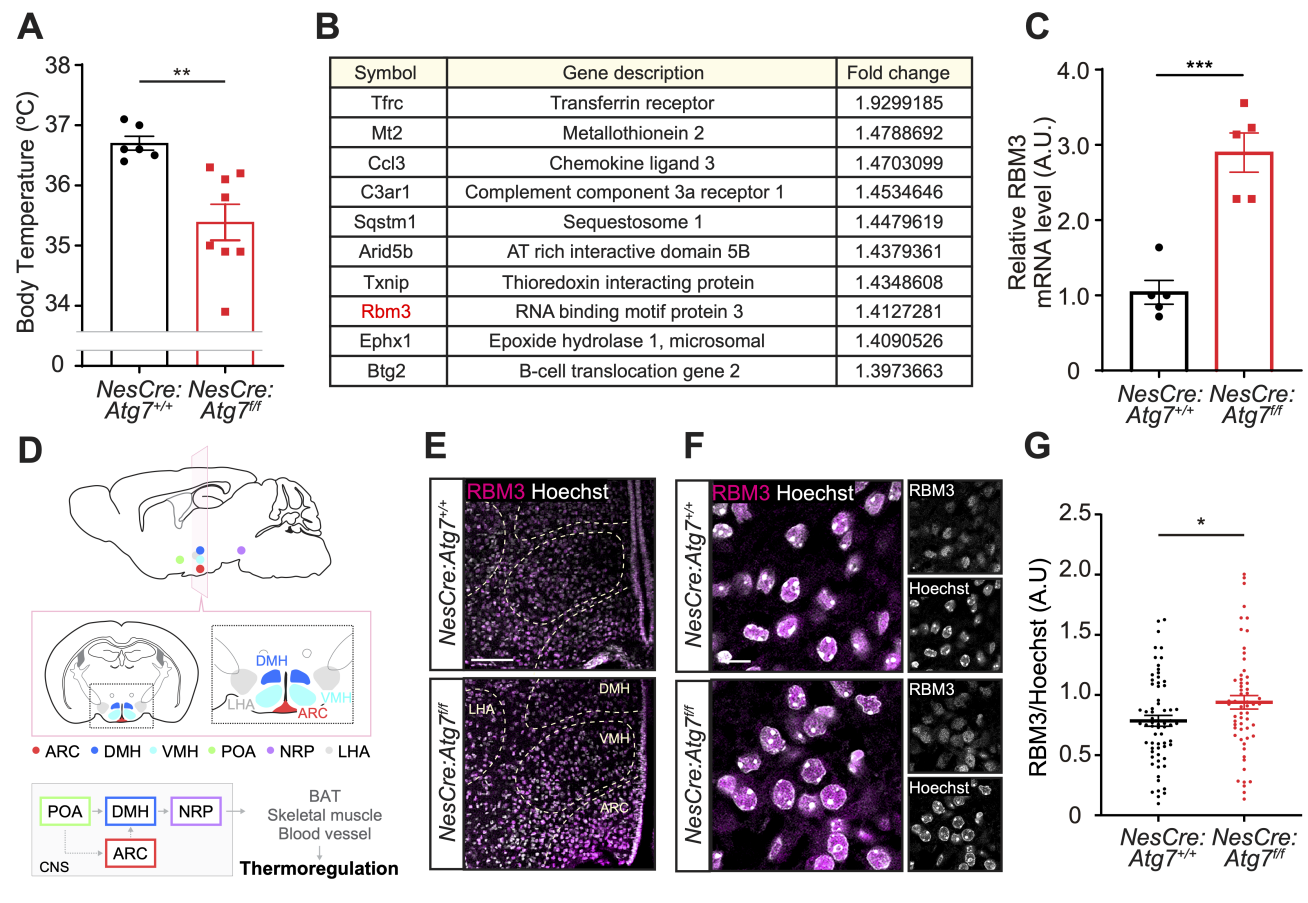
**(A)** Rectal temperature of the *Nestin-Cre* ; *Atg7 ^+/+^* and *Nestin-Cre* ; *Atg7 ^f/f^* mice (P21) at 10 a.m. ( *Nestin-Cre* ; *Atg7 ^+/+^* : n = 6, *Nestin-Cre* ; *Atg7 ^f/f^* : n=8). mean ± SEM. ^∗∗^ p < 0.01 by Student’s *t* -test. **(B)** Microarray analysis showing genes differentially expressed in *Nestin-Cre* ; *Atg7 ^f/f^* mice brain compared to *Nestin-Cre* ; *Atg7 ^+/+^* mice brain. The table indicates the upregulated genes in the brain of *Nestin-Cre* ; *Atg7 ^f/f^* mice. **(C)** RBM3 mRNAs were measured by quantitative RT-PCR analysis. The RNAs were isolated from mice brains (P21) and subjected to qRT-PCR analyses. The GAPDH levels were used for normalization. n=5; mean ± SEM; ***P<0.005 by Student’s *t* -test. **(D)** Schematic diagrams of the central circuit of the thermoregulation in the hypothalamus. ARC；the arcuate nucleus, DMH; the dorsomedial nucleus of the hypothalamus, VMH; the ventromedial hypothalamus, POA; the preoptic area, NRP; the nucleus raphe pallidus, LHA; the lateral hypothalamus area, BAT; brown adipose tissue. **(E)** Immunohistochemical analysis of *Nestin-Cre* ; *Atg7 ^+/+^* and *Nestin-Cre* ; *Atg7 ^f/f^* mice hypothalamus (P21). The brain cryosections were immunostained with anti-RBM3 antibody and Hoechst33342. Scale bar; 100 μm **(F)** Immunohistochemical analysis of *Nestin-Cre* ; *Atg7 ^+/+^* and *Nestin-Cre* ; *Atg7 ^f/f^* hypothalamus (P21). RBM3 is mainly localized in the nuclei. Scale bar; 20 μm **(G)** The quantification of RBM3 expression in the ARC. The graph indicates the ratio of RBM3 to Hoechst33342. *Nestin-Cre* ; *Atg7 ^+/+^* ; n=120 cells, *Nestin-Cre* ; *Atg7 ^f/f^* ; n=133 cells. 2 images obtained from one mice brain. This data is reproducible in at least two independent experiments. mean ± SEM; *P<0.05 by Student’s *t* -test.

## Description

It is known that autophagy controls energy and nutrient balance, contributing to regulating body temperature, circadian rhythm, and food intake (Kaur and Debnath, 2015). ATG7 is an essential protein that acts upstream of the autophagy cascade. It has been reported that neuron type-specific *Atg7* cKO mice exhibit distinct homeostatic phenotypes. For instance, a loss of *Atg7* gene in AgRP neurons in the hypothalamus causes abnormal energy balance and food intake (Kaushik et al., 2011). In addition, POMC neuron-specific *Atg7* cKO impairs neural projections to other nuclei, such as the paraventricular nucleus of the hypothalamus (PVN), the dorsomedial nucleus of the hypothalamus (DMH), and the lateral hypothalamic area (LHA) (Coupe et al., 2012), and showed a decrease in body temperature of approximately 0.5 ºC in the presence or absence of cold exposure (Martinez-Lopez et al., 2016). Notably, these hypothalamic neuron-specific *Atg7* cKO mice can survive in contrast to *Nestin-Cre* ; *Atg7 ^f/f^* cKO mice, usually die around one month of age (Komatsu et al., 2006). Although the biological relevance between autophagy deficiency and brain homeostasis has been investigated, the mechanisms that regulate body temperature in the postnatal stages have not been clarified. To examine this, we confirmed whether neural autophagy influences body temperature. To do this, we measured the rectal temperature in *Nestin-Cre* ; *Atg7 ^+/+^* and *Nestin-Cre* ; *Atg7 ^f/f^* mice [postnatal day (P) 21] in the morning. Consistent with the previous studies (Martinez-Lopez et al., 2016), the rectal temperature of *Nestin-Cre* ; *Atg7 ^f/f^* was lower at approximately 1.0 ºC than that of *Nestin-Cre* ; *Atg7 ^+/+^* mice, suggesting that autophagy is crucial for maintaining body temperature (Fig.1A). Next, to identify the specific target regulated by neural autophagy, we conducted the microarray analysis using *Nestin-Cre* ; *Atg7 ^+/+^* and *Nestin-Cre* ; *Atg7 ^f/f^* brains. The global gene expression pattern significantly changed in *Nestin-Cre* ; *Atg7 ^f/f^* mice compared to *Nestin-Cre* ; *Atg7 ^+/+^* (Fig.1B and Exon array dataset Table 1). We found that p62/SQSTM1 is a favorable target induced by autophagy deficiency. It is known that p62/SQSTM1 is upregulated in the *Nestin-Cre* ; *Atg7 ^f/f^* brain (Komatsu et al., 2007). In addition, the transferrin receptor is an intriguing target physiologically. It has been reported that loss of autophagy causes iron deficiency due to an inhibition of ferritin degradation (Hou et al., 2016). Because ferritin is an intracellular iron storage protein, abnormal autophagy causes the accumulation of ferritin-containing iron. Furthermore, we also identified that RBM3 is significantly upregulated in *Nestin-Cre* ; *Atg7 ^f/f^* brain in our transcriptome analysis (Fig.1B). It has been reported that RBM3 is one of the cold-inducible protein and regulates neurogenesis and local translation in neurons (Sertel et al., 2021; Xia et al., 2018; Zhu et al., 2019). Importantly, RBM3 is induced by body temperature drop, such as hibernation (Williams et al., 2005), and stabilizes the mRNA of circadian genes (Liu et al., 2013). Because the circadian rhythm is associated with the daily body temperature variation, these observations demonstrate that RBM3 is a potential candidate that links a defect in autophagy to lower body temperature. Then, we confirmed RBM3 gene expression in *Nestin-Cre* ; *Atg7 ^f/f^* brain. The mRNA levels in *Nestin-Cre* ; *Atg7 ^f/f^* brain were significantly increased compared to that in *Nestin-Cre* ; *Atg7 ^+/+^* brain (Fig.1C), indicating that loss of *Atg7* gene enhances RBM3 expression in the brain. Previous studies have reported that the preoptic area (POA) is a core region that governs body temperature (Kuraoka and Nakamura, 2011). It is known that the POA neurons project to the DMH, followed by regulating a body temperature via influencing the brown adipose tissue, skeletal muscle, and blood vessels. It is also known that the POA neurons project to the arcuate nucleus (ARC) and regulates the neural circuit in the hypothalamus implicated in the body temperature and circadian rhythm (Fig. 1D). Thus, we investigated which area RBM3 was expressed in the hypothalamus. RBM3 expression was ubiquitous; however, RBM3 expression in the hypothalamus was high in *Nestin-Cre* ; *Atg7 ^f/f^* brain. Especially, this phenomenon tended to be observed in the arcuate nucleus (ARC) in the hypothalamus (Fig. 1E). Interestingly, the subcellular localization of RBM3 was mainly localized in the nucleus (Fig. 1F), implying the possibility that RBM3 modulates the characteristics of target mRNA, such as splicing, stability, and export from the nucleus. In addition, the RBM3 level in the hypothalamic area of *Nestin-Cre* ; *Atg7 ^f/f^* mice was significantly increased compared to that in the same area of *Nestin-Cre* ; *Atg7 ^+/+^* mice (Fig. 1G). These results suggest that RBM3 regulates neuronal activity or regional homeostasis in the hypothalamic area, leading to the control of body temperature. Taken together, our data suggest that RBM3 is a potential mediator that underlies the regulation of body temperature associated with autophagy deficiency.

## Methods

**Microarray analysis**

*Nestin-Cre* ; *Atg7 ^+/+^* and *Nestin-Cre* ; *Atg7 ^f/f^* mice (P16) were euthanized using carbon dioxide. Their cerebral cortices were taken out using sterilized dissecting forceps and were then transferred into zirconia beads-containing tubes. After pulverizing and dissolving the brain with Isogen II (Nippon Gene) by shaking vigorously, the total RNAs were extracted according to the manufacturer’s instructions. The single-stranded cDNAs were generated from total RNA using an Ambion WT Expression kit (Ambion, Inc.), and labeled using a GeneChip WT Terminal Labeling and Hybridization kit (Affymetrix). These samples were incubated at 45°C for 17 h to hybridize on GeneChip mouse exon 1.0 ST arrays (Affymetrix). After hybridization, each probe array was washed and stained with GeneChip Fluidics Station 450 (Affymetrix) and scanned with an GeneChip Scanner 3000 (Affymetrix). Data were analyzed with GeneSpring 12.6 software (Agilent). The GEO accession number for the array data set is GSE214939.

**qRT-PCR**

Total RNAs from cerebral cortex were isolated by ISOGEN II (Nippon Gene) according to the manufacturer’s instructions. The cDNAs were synthesized by 100 units reverse transcriptase (ReverTra Ace, TOYOBO) together with 25 pmol random hexamer primer (TOYOBO), 20 nmol deoxynucleotide triphosphates (dNTPs), and 1.0 μg total RNAs. The quantitative reverse transcription PCR (qRT-PCR) was performed in 96-well plate (Thermo Fisher Scientific) using THUNDERBIRD SYBR qPCR mix (TOYOBO) in Thermal Cycler Dice Real Time System TP800 (TaKaRa). The relative quantity of the target expression was calculated by comparative threshold cycle (2 ^-ΔΔCT^ ) methods using Thermal Cycler Dice Real Time System Software (TaKaRa). This experiment was reproduced at least twice.

The following sequences were used as primers:

RBM3 forward, 5’-CAGATGCGATGAGAGCTATGAATGGAGAG-3’

RBM3 reverse, 5’-CTGGCAGACTTTCCTGCATGATCAACTC-3’

GAPDH forward, 5’- ACCACAGTCCATGCCATCAC-3’

GAPDH reverse, 5’- CACCACCCTGTTGCTGTAGCC-3’

**Immunohistochemistry and image processing**

*Nestin-Cre* ; *Atg7 ^+/+^* and *Nestin-Cre* ; *Atg7 ^f/f^* mice (P21) brains were perfused with 4% PFA-PBS after anesthesia and the brains were fixed with the same fixative solution overnight. After 30% sucrose-PBS infiltration, the samples were embedded in Tissue-Tek Optical Cutting Temperature compound (SAKURA) and sliced at 40 μm thickness using a cryostat (Leica Biosystems). The tissue sections were blocked for 1 h in 5% BSA in PBS. The samples were then incubated with primary antibody in 0.1% Triton X-100 in PBS (25 mM Tris–HCl [pH 7.5], 0.14 M NaCl) for 1 day (anti-RBM3 antibody, Proteintech, Cat#14363-1-AP, 1:1000) at 4 °C. Following the wash with PBS, the tissue sections were incubated with an anti-rabbit IgG Dylight Fluorochrome 594 (abcam) secondary antibody (1/1000) in 5% BSA-PBS together with 5.0 μg/ml Hoechst 33342 (Invitrogen) for 1h at room temperature. The tissues were mounted on the slides glass using VECTASHIELD Mounting Medium (Vector Laboratories). The samples were observed using a confocal laser scanning microscope (LSM700, Carl Zeiss) with 10× (Plan-Apochromat 10×/0.8 M27) and 40× (Plan-Apochromat 40×/1.3 Oil DIC M27) objective. The diode excitation lasers (Diode405, and Diode555) were operated and directed to a photomultiplier tube (LSM T-PMT, Carl Zeiss) through a series of band pass filters (Ch1:BP420-475 + BP500-610, Ch2:BP490-635, and Ch3:BP585-1000). RBM3 expression in the ARC was quantified using FIJI ImageJ software. The area of nuclear staining was binarized for setting the region of interest (ROI). The fluorescence intensities of RBM3 and Hoechst33342 in the ROI were calculated using the Analyze Particle function. The size of ROI (10-150 μm ^2^ ) was defined as a suitable area to extract the signals and to eliminate the noise and the aggregated cells in the meninges. All images were taken with the same setting (exposure time, signal amplification, objective lens). This experiment was repeated twice under the same condition.

**Measurement of rectal temperature**

The rectal thermometry of *Nestin-Cre* ; *Atg7 ^+/+^* and *Nestin-Cre* ; *Atg7 ^f/f^* mice (P21) was measured using a digital thermometer (SATO KEIRYOKI, SK-1260). Briefly, the thermometer was inserted into the rectum, followed by a measurement of the temperature. The thermometry was conducted at 10 am on time. This rectal temperature is corresponding to the core body temperature (Meyer et al., 2017). This experiment was reproduced at least three times.

**Mouse**

*Nestin-Cre* ; *Atg7 ^f/f^* mice are available from RIKEN BRC (RBRC02760) (Komatsu et al., 2006). Animal experiments were conducted according to the university guidelines for animal care and use and arrive guidelines. This study was approved by the Animal Experiment Committee at the University of Tsukuba (the approval numbers:19-340, 20-438, 21-443).

**Statistical analysis and data availability.**

Statistical significance was analyzed using Prism ver.6 software (GraphPad Software, Inc.). Adobe Creative Could CC (Photoshop 22.1.0 and Illustrator 25.0.1) and FIJI Image J 2.1.0/1.53c were used for all image processing.

## Extended Data

Description: Relative gene expression in the cerebral cortices (Nestin-Cre; Atg7+/+ mice vs Nestin-Cre; Atg7f/f mice ). Resource Type: Dataset. DOI: 10.22002/e6ha1-cg439
